# Faecal microbial diversity in a cattle herd infected by *Mycobacterium avium* subsp. *paratuberculosis*: a possible effect of production status

**DOI:** 10.1007/s11274-024-04080-1

**Published:** 2024-07-22

**Authors:** Wisal A. Elmagzoub, Sanaa M. Idris, Marwa H. E. Elnaiem, Mohamed E. Mukhtar, ElSagad Eltayeb, Sahar M. Bakhiet, Julius B. Okuni, Lonzy Ojok, Sulieman M. El Sanousi, Ahmed Abd El Wahed, Ahmed A. Gameel, Kamal H. Eltom

**Affiliations:** 1 Department of Animal Health and Safety of Animal Products, Institute for Studies and Promotion of Animal Exports, University of Khartoum, Shambat, 13314 Khartoum North Sudan; 2 Department of Biology and Biotechnology, College of Applied and Industrial Sciences, University of Bahri, Khartoum North, Sudan; 3 Department of Pathology, Faculty of Veterinary Medicine, University of Khartoum, Shambat, 13314 Khartoum North Sudan; 4 Department of Botany and Agricultural Biotechnology, Faculty of Agriculture, University of Khartoum, Shambat, 13314 Khartoum North Sudan; 5 Department of Agricultural Extension and Rural Development, Faculty of Agriculture, University of Khartoum, Shambat, 13314 Khartoum North Sudan; 6 Faculty of Medicine, Al Neelain University/Ibn Sina Specialised Hospital, Street 17-21, Alamarat, 12217 Khartoum Sudan; 7 Department of Molecular Biology, Institute of Endemic Diseases, University of Khartoum, Khartoum, Sudan; 8 College of Veterinary Medicine, Animal Resources and Biosecurity (COVAB), Makerere University, P. O. Box 7062, Kampala, Uganda; 9 Department of Pathology, Faculty of Medicine, Gulu University, P.O.Box 166, Gulu, Uganda; 10 Department of Microbiology, Faculty of Veterinary Medicine, University of Khartoum, Shambat, 13314 Khartoum North Sudan; 11 Faculty of Veterinary Medicine, Institute of Animal Hygiene and Veterinary Public Health, Leipzig University, An den Tierkliniken 1, 04103 Leipzig, Germany

**Keywords:** *Mycobacterium avium* subsp. *paratuberculosis*, MAP, Microbiome, Production status

## Abstract

*Mycobacterium avium* subsp. *paratuberculosis* (MAP) causes Johne’s disease, or paratuberculosis (PTB) in ruminants, besides having zoonotic potential. It possibly changes the gut microbiome, but no conclusive data are available yet. This study aimed at investigating the influence of MAP on the faecal microbiome of cattle naturally infected with PTB. In a follow up period of 10 months, PTB status was investigated in a herd of dairy cattle with history of clinical cases. Each animal was tested for MAP infection using serum and milk ELISA for MAP anti-bodies and IS900 real-time PCR and recombinase polymerase amplification assays for MAP DNA in the faeces and milk monthly for 4 successive months, then a last one after 6 months. The faecal samples were subjected to 16S rDNA metagenomic analysis using Oxford Nanopore Sequencing Technology. The microbial content was compared between animal groups based on MAP positivity rate and production status. All animals were MAP positive by one or more tests, but two animals were consistently negative for MAP DNA in the faeces. In all animals, the phyla firmicutes and bacteroidetes were highly enriched with a small contribution of proteobacteria, and increased abundance of the families *Oscillospiraceae*, *Planococcaceae*, and *Streptococcacaceae* was noted. Animals with high MAP positivity rate showed comparable faecal microbial content, although MAP faecal positivity had no significant effect (*p* > 0.05) on the microbiome. Generally, richness and evenness indices decreased with increasing positivity rate. A significantly different microbial content was found between dry cows and heifers (*p* < 0.05). Particularly, *Oscillospiraceae* and *Rikenellaceae* were enriched in heifers, while *Planococcaceae* and *Streptococcaceae* were overrepresented in dry cows. Furthermore, abundance of 72 genera was significantly different between these two groups (*p* < 0.05). Changes in faecal microbiome composition were notably associated with increasing MAP shedding in the faeces. The present findings suggest a combined influence of the production status and MAP on the cattle faecal microbiome. This possibly correlates with the fate of the infection, the concern in disease control, again remains for further investigations.

**Supplementary Information:**

The online version contains supplementary material available at 10.1007/s11274-024-04080-1.

## Introduction

Infection by *Mycobacterium avium* subsp. *paratuberculosis* (MAP) causes paratuberculosis (PTB) or Johne’s disease (JD) a chronic untreatable disease in ruminants besides many other species. It is a notifiable disease that impacts animal welfare and economy (Donat et al. 2020). Also, MAP is implicated in Crohn’s disease (CD) in human (Duffy and Behr 2020) and has been linked to many other chronic conditions (Elmagzoub et al. 2022). This suspected link points out MAP as a public health threat and raises the safety issue of dairy and meat products (Balseiro et al. 2019; Garvey 2020; Dow 2015).

The main route of MAP transmission is the oral- faecal route; therefore, the key control is the shedding animal(s) (Moreira et al. 2019). PTB control programmes were set as early as 1920s (Donat et al. 2020), but there are only two reports about eradication of the disease (Fichtelova et al. 2022; Whittington et al. 2019). Young calves are more vulnerable to PTB, but the reason behind their susceptibility to the infection remains unknown. Immature immune system and impaired intestinal barrier integrity are among the hypothesized reasons (Fecteau 2018; Mortier et al. 2015).

The microbial community in the gut, the microbiome, is essential for improving feed efficiency, for health maintenance and for resistance to diseases (Clemmons et al. 2019). It was found to guide the development of the immune system and gut epithelium (Lu and Claud 2019; Takiishi et al. 2017) and plays an essential role in many physiological processes (Clemente et al. 2018). It also represents a complementary part of the mature digestive system in ruminants (Amin and Seifert 2021).

Previous microbiome studies investigated faecal microbiome in ruminants found firmicutes and bacteroidetes are the most abundant phyla. Among others, proteobacteria and actinobacteria were found in different proportions beside verrucomicrobia, tenericutes. The commonly assigned families in faecal samples were *Bacteroidaceae*, *Ruminococcaceae Lachnospiraceae*, *Prevotellaceae*, *Rikenellaceae*, *Planocococcaceae* and *Christensenellaceae* with their lineages (Zhang et al. 2022; Dias and Ametaj 2017; Aricha et al. 2021; Alipour et al. 2018; Wang et al. 2022; Zhao et al. 2022; Dill-McFarland et al. 2017; Haley et al. 2020).

Many factors affect the microbiome composition, some of which are key influencers throughout the development period such as gestation, genetic or inheritance of dam’s microbiome, feeding strategy and weaning (Arshad et al. 2021). Other factors could predispose to transient dysbiosis in mature microbiome such as health conditions, physiological state, and diet (Russell and Rychlik 2001; Weimer et al. 2010; Henderson et al. 2015; Holman and Gzyl 2019). For instance, reproductive hormones are known to have bidirectional effect on the gut flora (Hussain et al. 2021). They cause microbial shift attributed to a physiological state, such as pregnancy. Enrichment with *Bifidobacterium* was provided as a model for the effect of progesterone (Nuriel-Ohayon et al. 2019) the dominating hormone during pregnancy. In addition, age related difference was reported in a study that compared the microbiome content from 2-weeks of age to first lactation and a significant difference between age groups was found (Dill-McFarland et al. 2017).

For ruminants, gut microbes are essential for food digestion and feed efficiency, as they digest complex carbohydrates producing short chain fatty acids (SCFAs) (Al Bander et al. 2020). These compounds have important functions including the formation of rumen papillae (Ragionieri et al. 2016). Moreover, microbiome can prevent gastrointestinal infection through colonisation resistance (Amin and Seifert 2021), and it can also protect against gut inflammation such as that induced by MAP (Arshad et al. 2021; Matthews et al. 2021; Zeineldin et al. 2018). The difference in the microbiome composition at the time of infection was supposed as a factor for inter-individual variation in the disease course (Mortier et al. 2015), hence, it can be targeted to decrease infection burden. Therapeutic manipulation of microbiota had shown fruitful outcomes; *Dietzia* subsp. C79793-74 was used to reconstitute the gut flora in naturally infected cattle, and it succeeded in mitigating the infection symptoms and signs, even more, some were cured (Click and Kampen 2010). Further, *Dietzia* subsp. 79,793-74 was found to prevent development of JD when used in calves of infected dams (Click 2011). Administration of *Lactobacillus animalis* to a murine model of MAP infection was found to modulate the inflammatory mediators (Karunasena et al. 2013).

On the other hand, the potential of MAP to change microbiome composition leading to dysbiosis has been barely studied. When the microbiome of infected animals was compared with that of a control (non-shedding) group, an increase in the abundance of the genus *Psychrobacter* with a decrease in that of *Oscillospira*, *Ruminoccocus* and *Bifidobacterium* genera was observed in MAP shedding animals (Kaevska et al. 2016). Another study compared naturally infected cows (MAP+ve), MAP−ve herd mates (exposed) and a third group from a MAP−ve herd. In the MAP+ve group, an increased abundance of the genera *Psychrobacter*, *Bacillus* and *Enterococcus* with a decrease in the genera belonging to the families *Clostridiaceae*, *Paraprevotellaceae*, *Rikenellaceae* and *Bacteroidaceaece* was observed (Fecteau et al. 2016). Other investigators noted in experimentally infected calves an increase in *Planococcaceae* and *Paraprevotellaceae* with a decrease in genera belonging to the families *Ruminococcaceae* and *Akkermansiaceae* (Derakhshani et al. 2016). These studies (Kaevska et al. 2016; Fecteau et al. 2016) used only one test and once to investigate for MAP infection and to classify the animals under study. It is noteworthy that most of the tests used for detection of MAP infection have insufficient sensitivity (Barkema et al. 2018), since PTB is a chronic disease and the course of the disease is long from the infection to the development of the clinical disease and the sensitivity of a test affected by the stage of the disease. In the early stages, animals either do not shed MAP in the faeces or the shedding is intermittent and therefore, culture, the gold standard, would give false negative results. The same molecular testing for MAP in the faeces is affected by the intermittent shedding. Also, the immune response in the early stages is cell mediated, therefore antibody-based tests such as ELISA are insensitive, therefore, neither one test nor a single examination can confirm MAP negativity. Also, the other study (Derakhshani et al. 2016) investigated the microbiome in experimentally infected animals. However, experimental infection is not as similar as natural infection (Mortier et al. 2015). Taking into account these observations, in this study different tests and multiple samples were used to investigate the status of MAP infection in dairy cattle herd with history of clinical cases of PTB. Thereafter, the faecal microbiome was investigated and the animals were compared according to MAP positivity in the faeces, the production status.

## Materials and methods

### Description of the study farm

The study targeted a dairy cattle herd (25–30 heads) in a farm located in Khartoum North, the Sudan. The herd was composed of 16 milking cows in age range 2–10 y, 8 heifers in age range 1–2 y and 2 calves (< 1y). In this farm, there was a complaint of chronic diarrhoea with reduced body condition and milk production as a third case in the farm; the first two cases died a few years ago and they were clinically diagnosed as having JD, but without laboratory test confirmation. This third case deteriorated within 1 month from the first visit, besides milk production decreased sharply and the animal became emaciated with shooting diarrhoea typical to an advanced stage of JD. Faecal sample obtained from this case was cultured as performed before (Idris et al. 2022).

Based on the clinical diagnosis and the later isolation of MAP from the third case, PTB was considered established on the farm. The herd was followed-up to investigate the infection status and then to conduct the faecal microbiome study.

### Detection of *Mycobacterium avium* subsp. *paratuberculosis* infection and categorization of the animals for the microbiome study

#### Samples collection

Samples (faeces, milk, and blood) were collected monthly for 4 months, and a fifth collection was conducted after 6 months from the last collection. All samples were collected by licensed personnel.

The blood was obtained from the jugular vein in a sterile plain vacutainer after disinfecting the area. Disposable obstetric gloves were used for collection of faecal samples directly from the rectum to avoid cross-contamination. Milk samples were collected aseptically from all teats of lactating cows, those in the dry period were excluded in some collection sets. The serum, faeces and milk samples were kept at − 20 °C until processing.

#### Detection of anti-MAP antibodies

The harvested sera were tested for the presence of anti-MAP antibodies using IDEXX ELISA kits (Westbrook, ME, USA), following the manufacturer’s protocol. Briefly, the test samples and the kit controls were diluted with the provided buffer containing *Mycobacterium phlei* and pre-incubated for 35 min at room temperature (RT). A total of 100 µl of diluted samples and the controls were added to microtitre plate wells coated with MAP antigen and then incubated at RT for 45 min. The plates were then washed thrice. This was followed by addition of 100 µl of conjugate to each well, then the plates were incubated for 30 min at RT and then were washed thrice. Next, 100 µl of tetramethylbenzidine (TMB) solution was added and the plate was incubated again at RT for 10 min followed by addition of stop solution (100 µl). The optical density of the plates was read at 450 nm using a spectrophotometer (Plate reader, DAS, Palombara Sabina—Italy).

#### Processing of faecal samples

First, ~ 0.5 g of faeces was suspended in 5 ml distilled water and left till coarse matter settled down, then the supernatant was transferred to a new Eppendorf tube and centrifuged at 6000×*g* for 10 min. The sediment was used to prepare inoculum for culture and DNA extraction.

#### Extraction of DNA from the faeces

Using QIAamp DNA Blood Mini Kit (Qiagen, Hilden, Germany), the DNA was extracted from faeces following the manufacturer instructions. Briefly, the sediment from the processed faecal samples was re-suspended in 100 µl PBS and 50 µl of 20% sodium dodecyl sulphate, homogenized for 4 min in Intelli-Mixer’s™ (ELMI, Riga, Latvia) and incubated at 37 °C for 30 min, then 200 µl lysis buffer was added and incubated at 37 °C for 30 min. Proteinase K (60 µl) and additional 600 µl lysis buffer were added and incubated at 56 °C followed by 95 °C for 30 min and 15 min, respectively. Purification was performed using the silica membrane column after addition of 400 µl absolute molecular grade alcohol. The retained DNA in the membrane was eluted by 100 µl elution buffer.

#### Extraction of DNA from milk

For better isolation of DNA from milk the method developed before (Gao et al. 2007) was followed. Briefly, the samples were heated at 95 °C for 10 min, centrifuged at 3500×*g* for 30 min and the whey layer was removed carefully. The mixed pellet and cream layer were re-suspended in 15 ml of 0.75% hexadecyl pyridinium chloride and incubated at 22 °C for 30 min. After centrifugation at 2000×*g* for 15 min, the liquid phase including the cream layer was carefully discarded and the pellet was used for DNA extraction using QIAamp DNA Blood Mini Kit (Qiagen), as the same as faeces, but with a single lysing step.

#### Recombinase polymerase amplification (RPA)

An RPA assay targeting the MAP IS900 developed earlier (Hansen et al. 2016) was used to test extracted DNA from faeces and milk. The reagents of the assay in this study were supplied by Jiangsu Qitian Gene Biotechnology Co., Ltd** (**Jiangsu, China). Briefly, the reaction mix was composed of 5 µl DNA template, 29.5 μl rehydration buffer, 2.1 µl (10 p mol/μl) of each forward (5′-CGTGGACGCCGGTAAGGCCGACCATTACTGCATGG-3′) and reverse primer (5′-CGCCGCAATCAACTCCAGCAGCGCGGCCTC-3′) with 2 µl (10 p mol/μl) exo probe (5′-ACGCCGGTAAGGCCGACCATTACTGCATGGT BHQ1-dt, Tetrahydro-furan and Fam-dTTAACGACGACGCGCA-PH-3′) and 2.5 µl of Mg acetate. The tubes were incubated at 42 °C in the tube scanner (TwistDx Ltd., Cambridge, UK) for 20 min.

#### Real-time polymerase chain reaction (real- time PCR)

The IS900 real- time PCR assay for MAP described previously (Münster et al. 2013), was performed in T-Optical Real Time PCR Thermal Cycler (Biometra, Analytik Jena, Jena, Germany). Briefly the reaction mixture was 10 µl Qiagen Master mix, 0.5 µl of each 10 pmol/μl) FP: 5′-TACCGCGGCGAAGGCAAGAC-3′ and RP: 5′-CGGAACGTCGGCTGGTCAGG-3′, 1 µl of 10 pmol/μl probe: 5′-FAM-ATGACATCGCAGTCGAGCTG-BHQ-1–3 and 3 µl molecular biology grade H_**2**_O in addition to 5 µl of the DNA template. The thermal cycler was programmed as follows: first 10 min pre-incubation at 95 °C, then 40 cycles of 95 °C for 15 s, 55 °C for 30 s and 72 °C for 35 s followed by a final cooling step at 40 °C for 30 s.

### Faecal bacterial community profiling by 16S rDNA amplicon sequencing

#### Extraction of DNA from faeces

The aliquots from the fifth collection of faecal samples (stored at − 20 °C) were used, first thawed at room temperature then used for DNA extraction. The MagMax Microbiome Ultra Nucleic Acid Isolation Kit (Life Technologies, Austin, USA) was used as instructed by manufacturer with slight modifications. Briefly, ~ 100 mg of faeces was added to 800 µl lysis buffer in the supplied beads- containing tubes, homogenized in the Intelli-Mixer’s™ (ELMI) for 30 min. After centrifugation at 14,000×*g* for 2 min, the supernatant was purified by mixing with 520 µl of binding beads solution (500 µl buffer + 20 µl magnetic beads) for 15 min. After short spin, the beads were pelleted in the magnet rack; the pellets were washed twice using 1 ml of the washing solution and followed by 1 ml of 70% molecular grade alcohol twice. After evaporation of the remaining alcohol, the pellets were re-suspended in 50 µl elution buffer and incubated at 75 °C for 5 min and pelleted again. The concentration of the DNA was measured using Qubit 4 dsDNA BR Assay kit (Thermo Fisher Scientific, Waltham, MA, USA) and used for metagenomic analysis.

### Metagenomic analysis of the DNA

#### Amplification of the 16S rDNA

The 16S Barcoding Kit (SQK-RAB204) and protocol supplied by Oxford Nanopore Technologies (ONT) (Cambridge, UK) were used to amplify ~ 1500 bp of the 16S rRNA gene, using 10 ng genomic DNA, 1 µl barcode, each containing the primers 27F 5′-AGAGTTTGATCCTGGCTCAG-3′ and 1492R 5′-GGTTACCTTGTTACGACTT-3′, with 25 µl of LongAmp Taq 2 × Master Mix (M0287) (New England Biolabs, Ipswich, MA, USA) and nuclease free water for up to 50 µl reaction mix. The PCR programme was as follows: initial denaturation step for 1 min at 95 °C, then 25 cycles each composed of 20 s at 95 °C, 30 s at 55 °C and 2 min at 65 °C, followed by a final extension for 5 min at 65 °C.

The PCR products were purified by 30 µl Agencourt AMPure XP beads (Beckman Coulter Inc, CA, USA) and eluted by 10 µl of Tris–HCl buffer (10 mM Tris–HCl pH 8.0 with 50 mM NaCl). The eluates were pooled, and from this mix 10 µl was used for DNA library preparation.

#### DNA library preparation and sequencing

First, the DNA library was prepared by incubating the pooled eluates of PCR products with 1 ml adapter at room temperature for 5 min. The priming solution (30 µl flushing tether added to a tube of flushing buffer) was loaded in the flow cell. After removing 20–30 µl from the flow cell’s storage solution through the priming port to ensure the absence of air bubbles, ~ 800 µl then ~ 200 µl of the priming solution were added with 5 min interval.

A mix of 25.5 µl of the loading beads, 34 µl of the sequencing buffer, 11 µl of the DNA library and 4.5 µl nuclease- free water was loaded carefully into the flow cell through the Spot-On port.

The sequencing was initiated in the MK1C (ONT) device using flow cell R 9.4, and the time was set to 12 h. The integrated software, MinKNOW, performed the data acquisition task and base calling, through Guppy, generating the Fast5 and Fastq files.

#### Analysis of the bacterial community composition

The retrieved data from the MK1C, the Fastq files, were uploaded in the cloud-based analysis service of the ONT, the EPI2ME, through the desktop agent. The 16S workflow was used with quality score 10, minimum length 1500 bases and BLAST E-value of 0.01. The programme aligns the sequencing reads to the NCBI 16S bacterial database and provides taxonomic classification up to the species level with the number of reads of each taxon. For comparison purposes, operational taxonomic units (OTUs) counts were standardized by counting the relative abundance and then multiplied by the median sample read depth using phyloseq package (McMurdie and Holmes 2013). The standardized data were merged at genus level using a modified tax-glon function of the same package. In the downstream analysis, all taxa were considered in the analysis except those present at very low numbers in a minority of samples. The data were filtered upon two criteria: the abundance and/ or frequency (kviljoen LK. Microbiome_custom_functions.R 2023). That is, taxa included in the analysis are those found in at least 10 counts in at least 20% of the samples, and/ or those have a relative abundance of ≥ 1% of the total number of reads.

For the observed number of taxa, Pielou (evenness) and Shannon (Richness) indices were used to study the bacterial community of each animal and to measure the diversity within the samples (alpha diversity), using the estimate-richness function of phyloseq R package (R Foundation, Vienna, Austria).

For measuring the difference between samples (beta diversity) Bray–Curtis dissimilarity measure and the non-metric multidimensional scaling (NMDS) as methods in phyloseq package were used (47).

### Statistical analysis

Binary logistic regression model was used to obtain logical inferences about the relationships between MAP positivity and the factors: age, animal source, production status, milk production and body condition (SPSS Statistics for Windows, version x. 23 (SPSS Inc., Chicago, Ill., USA).

Multiple hypotheses testing with False Discovery Rate (FDR) was performed using negative binomial distribution (glm.edgeR function in edgeR package) to investigate for significant association between taxa and categorization criteria.

## Results

By the end of the experiment, out of 26 cows in the herd 22 cows had completed the collection period and were investigated for MAP infection parameters: 12 milking cows, 3 dry, 6 heifers and 1 calf. They showed varying positivity rate in MAP infection tests, however all of them were positive for one of the tests except one, a heifer.

### Determination of infection status

#### Detection of anti-MAP antibodies

Antibodies against MAP were detected in the serum of 22.7% (5 out of 22) of the animals in the age range 5–10 years in the first visit. In the following visits, either only one cow was ELISA positive or none. In the succeeding, with the exception of one animal that was consistently positive, and another one that was positive once visit none of the sampled animals was positive in ELISA. Similarly, 20% (3 out of 15) of the animals sampled in the first visit were positive in milk ELISA, while in the following visits, only one cow was positive continuously (Supplementary File 1, Table 1).

#### Determination of MAP shedding in the faeces and milk

**Fig. 1:**
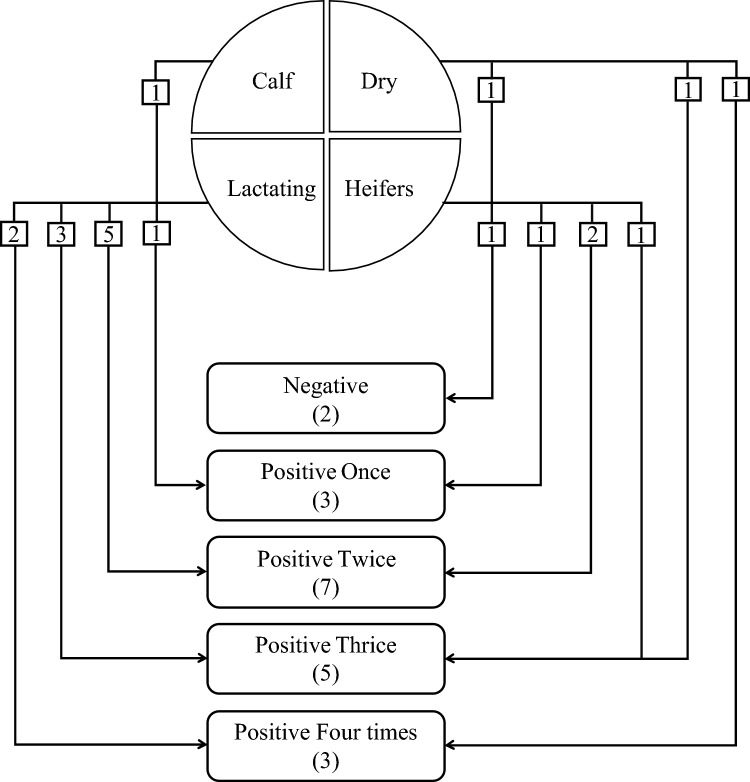
Categorization of the animals in the study farm based on detecting *Mycobacterium avium* subsp. *paratuberculosis* DNA in faeces throughout the five visits; the frequency of positivity in faeces and based on production status, the figures indicate the number of animals

Throughout the follow-up period, MAP DNA was detected in all faecal and milk samples, except one cow which was consistently negative all over the sampling period in all sample types (faeces and milk); the results were interpreted as absolute negative and positive. In the faecal samples, all animals were positive in at least one visit except two were consistently negative for MAP DNA and the remaining animal(s) did not give consecutively positive results, accordingly, in most animals, the shedding of MAP was inconsistent (Fig. 1).

Using both molecular assays, (real-time PCR and RPA) MAP DNA in the faeces was detected in 45.5% of the animals in the first sampling; the peak was 65.2% in the second sampling and decreased to 34.8 and 22.7% in the two successive samplings, respectively. In the last sampling, MAP DNA was not detected in all faecal samples (Supplementary File 1, Table 2).

In milk, MAP DNA was detected in 53.8% of the samples obtained in the first sampling and decreased sharply to 18.2% in the second sampling, but increased to 63.6 and 41.7% in the third and fourth samplings, respectively. In the last sampling, it decreased again to 9.1% (Supplementary File 1, Table 2). The statistical analysis showed no correlation between any of the animal’s factors (age, animal source, production status, milk production, and body condition) and MAP positivity. Animals were categorized into groups based on different criteria: the production status and the test results of detecting MAP in the faeces: the DNA positivity and frequency of MAP DNA positivity. Alongside, within each category the animals were grouped into three subgroups based on the age, viz; < 2y, 2–5 y and 5–10.

### Faecal microbial composition

#### Abundance of different taxa

A total of 22 animals were included in the microbiome study, but samples from two animals viz a calf (35061) and a milking cow (350100) did not provide sufficient sequence data, therefore, were excluded from the analysis. The remaining 20 animals were 11 milking cows, 3 dry cows, 5 heifers and 1 calf of 9 months old.

Overall, 17 bacterial phyla, 39 families, 94 genera and 210 species were assigned in the faecal samples. At the species level, the minimum number of observed taxa was 75 in the calf and the highest number was 195 species in milking cow/dry cow/heifer.

**Fig. 2:**
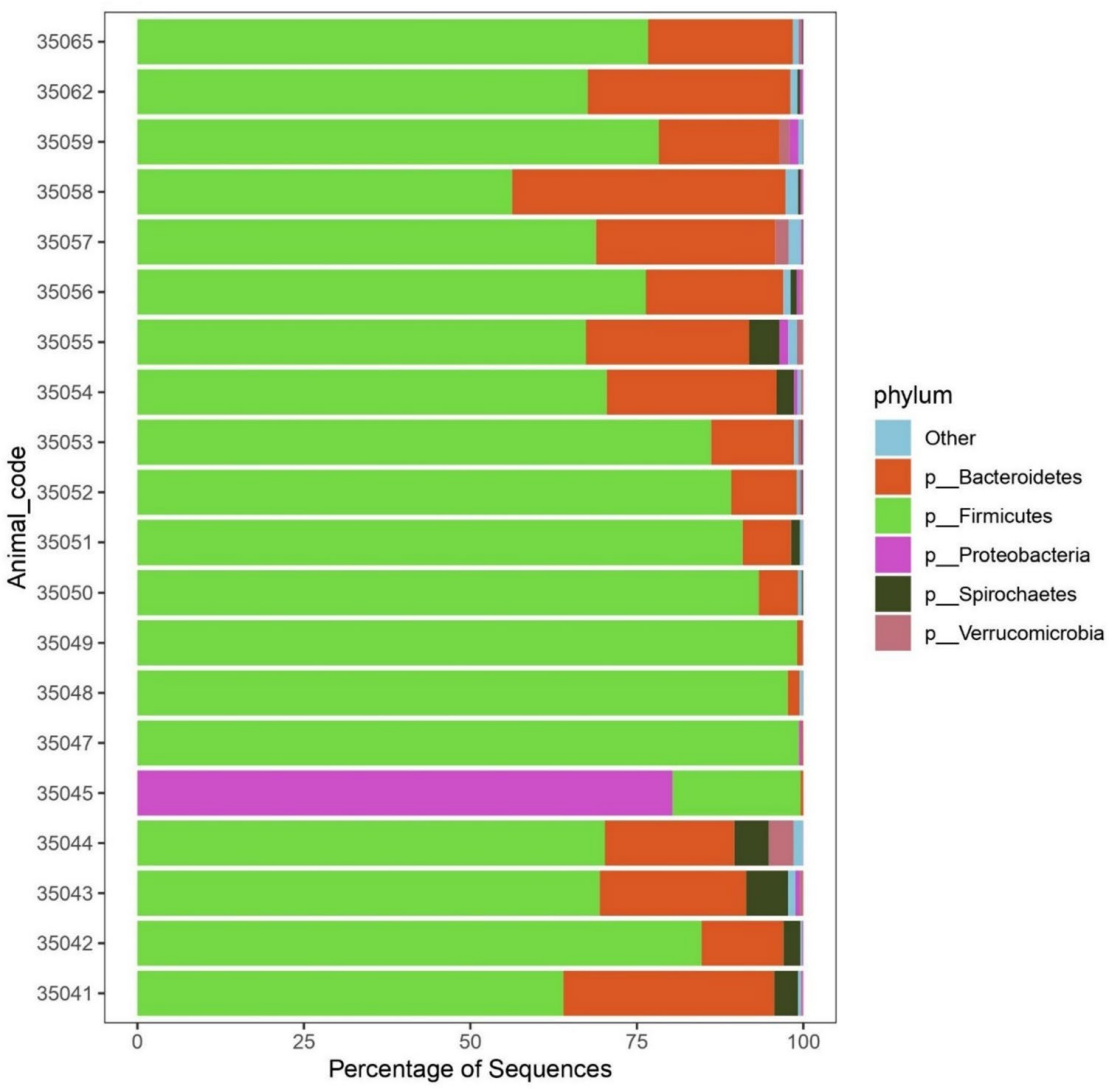
The relative abundance of bacterial phyla in the faecal samples of cattle in a dairy farm with history of paratuberculosis

In nearly all samples (Fig. 2), the firmicutes was the most abundant phylum (19.5–99.2%) followed by bacteroidetes (0.2–80%). The phyla proteobacteria, spirochetes and verrucomicrobia were found in some samples as minimum in abundance as < 0.1% and their highest abundance was 6%, 1.5% and 1.8, respectively.

Also, among the 39 assigned families; the average relative abundance (ARA) ranked *Oscillospiraceae* as the most abundant family, however it was dominating in 9 out of the 20 animals. *Planococcaceae* followed and predominated in 4 animals by ≥ 30% abundance in each. *Streptococcaceae* was the most abundant family in 3 animals only, but reached up to 88% of the families present in one of them. These families together with *Clostridiaceae* and *Rikenellaceae* represented > 60% of the families in each sample.

At the genus level, the collective abundance of the top 10 genera represented ~ 60% of those assigned. *Streptococcus* had the highest ARA by 10.3%, despite it depleted in some animals (N = 4) in the remaining animals it was in abundance range 0.1–87.3%. *Clostridium*, the second in abundance, was the only genus that was found in all animals: in range 0.8–25.7%. *Solibacillus* was the third in order despite being < 1% in 11 animals. *Oscillobacter*, *Alistipes*, *Bacteroides* and *Acetivibrio* were found in the same animals (N = 16) and were depleted in the others. *Comamonas* was found in only one animal, but in high abundance (59.4%).

Out of the 210 assigned species, only 25 of them showed relative abundance of ≥ 1% and 99 showed abundance of ≥ 0.1%. The top 10 abundant species represented 44% of the total abundance of the assigned species. *Streptococcus lutetiensis* was the most abundant species showing 8% ARA, followed by *Solibacillus isronensis* (7.4%) and *Oscillibacter valericigenes* (6.3%). Despite the ARA of *Rummeliibacillus pycnus* was 5.1%, it was not assigned in 8 animals, and showed abundance > 1% in only 3 animals. Although, *Kurthia massiliensis* was found in only one animal it showed ARA 4.4%.

**Fig. 3:**
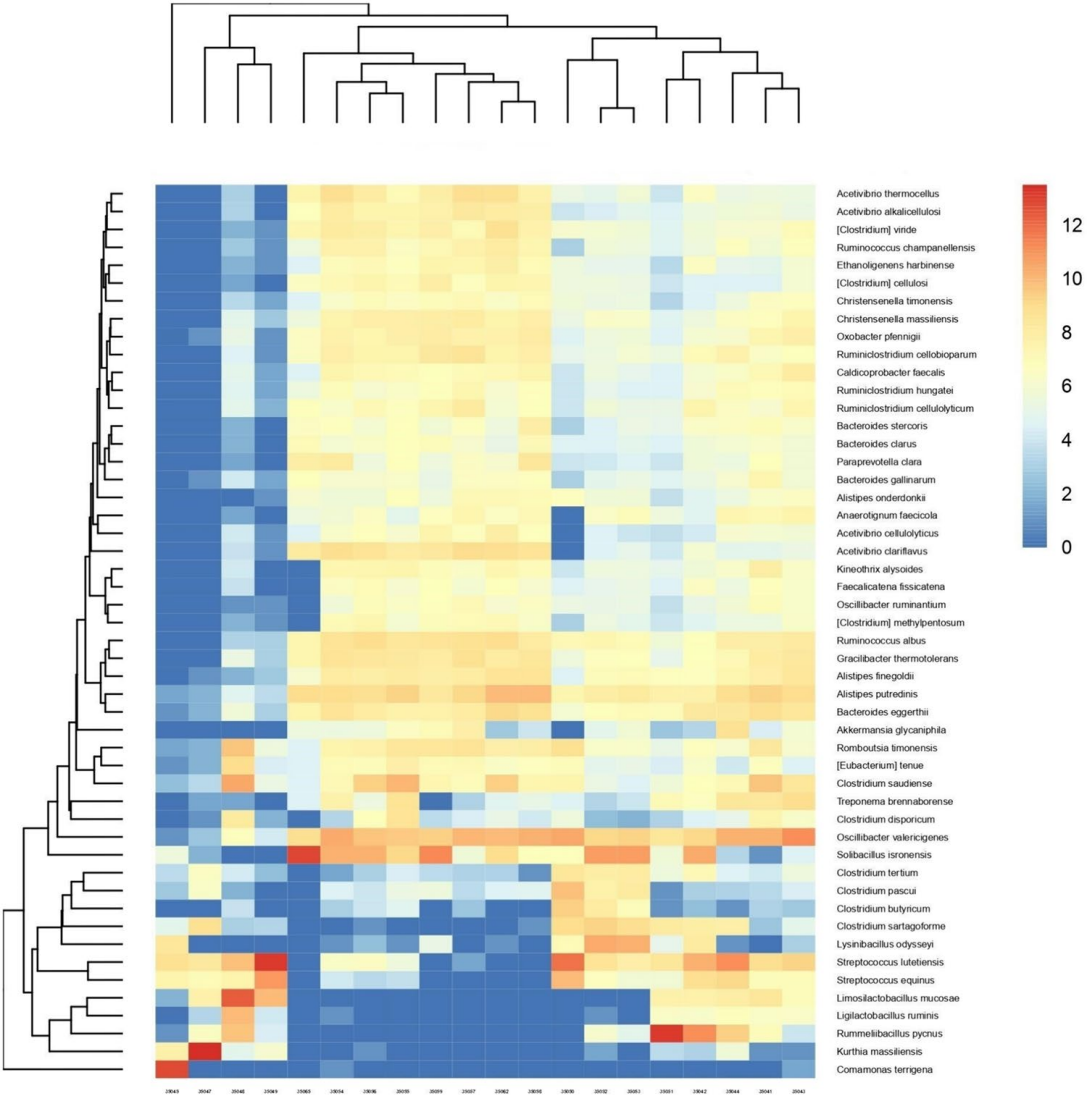
Heatmap showing the differential abundance of the top fifty species in the faecal samples of cattle in a dairy farm with history of paratuberculosis

In the differential abundance of the top 50 species in the samples from individual animals (Fig. 3); the animals clustered into groups based on the similarity in composition. A group of four animals (35045, 35047, 35048 and 35049) was highly characterised by diminishing of some taxa (e.g., *Bacteroides*, *Paraprevotella*, *Ruminococcus*, and *Oscillibacter*) compared with the remaining animals. On the other hand, compared with the others, in each one of these four animals one species was highly enriched viz *Kurthia massiliensis* in 35,047, *Comamonas teriigena* in 35,045, *limosilactobacillus mucosae* in 35,048 and *Streptococcus lutetiensis* in 35049). The remaining animals clustered into two groups; one comprised the samples from the animals: 35065, 35054, 35056, 35055, 35059, 35059, 35057, 35062 and 35058, and the other group included the samples from animals 35065, 35054, 35056, 35056, 35059, 35057, 35056 and 35058 each of comparable composition. These groups shared the relatively high abundance of *Oscillibacter valericigenes*, especially when compared with the four animals.

#### Bacterial community diversities among animals and groups

**Fig. 4:**
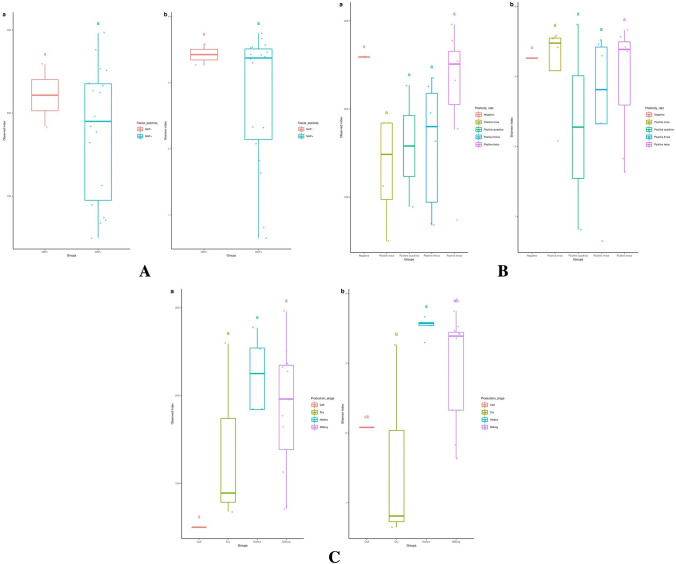
**a** Diversity of bacterial communities in the faeces of cattle investigated for *Mycobacterium avium* subsp. *paratuberculosis* (MAP) measured by observed and Shannon indices of animals with different DNA detection in faeces. **b** Diversity of bacterial communities in the faeces of animals investigated for *Mycobacterium avium* subsp. *paratuberculosis* measured by observed and Shannon indices for groups of animals with varying frequency of DNA detection in faeces. **c** Diversity of bacterial communities in the faeces of animals investigated for *Mycobacterium avium* subsp. *paratuberculosis* measured by observed and Shannon indices for groups of animals based on production status

In each sample the alpha diversity indices, Shannon (richness) and Pielou (evenness), inconsistently increased with the increase in number of observed taxa. However, animals (N = 2) with the lowest number of observed taxa also showed the lowest Shannon and Pielou indices. Across groups outlined in Fig. 1, viz; MAP+ve (18) vs MAP−ve (2), animals with varying positivity rate, and animals at different production status; the alpha diversity analysis calculated by Shannon indices, revealed a significant difference between heifers and dry cows (Fig. 4a–c).

**Fig. 5:**
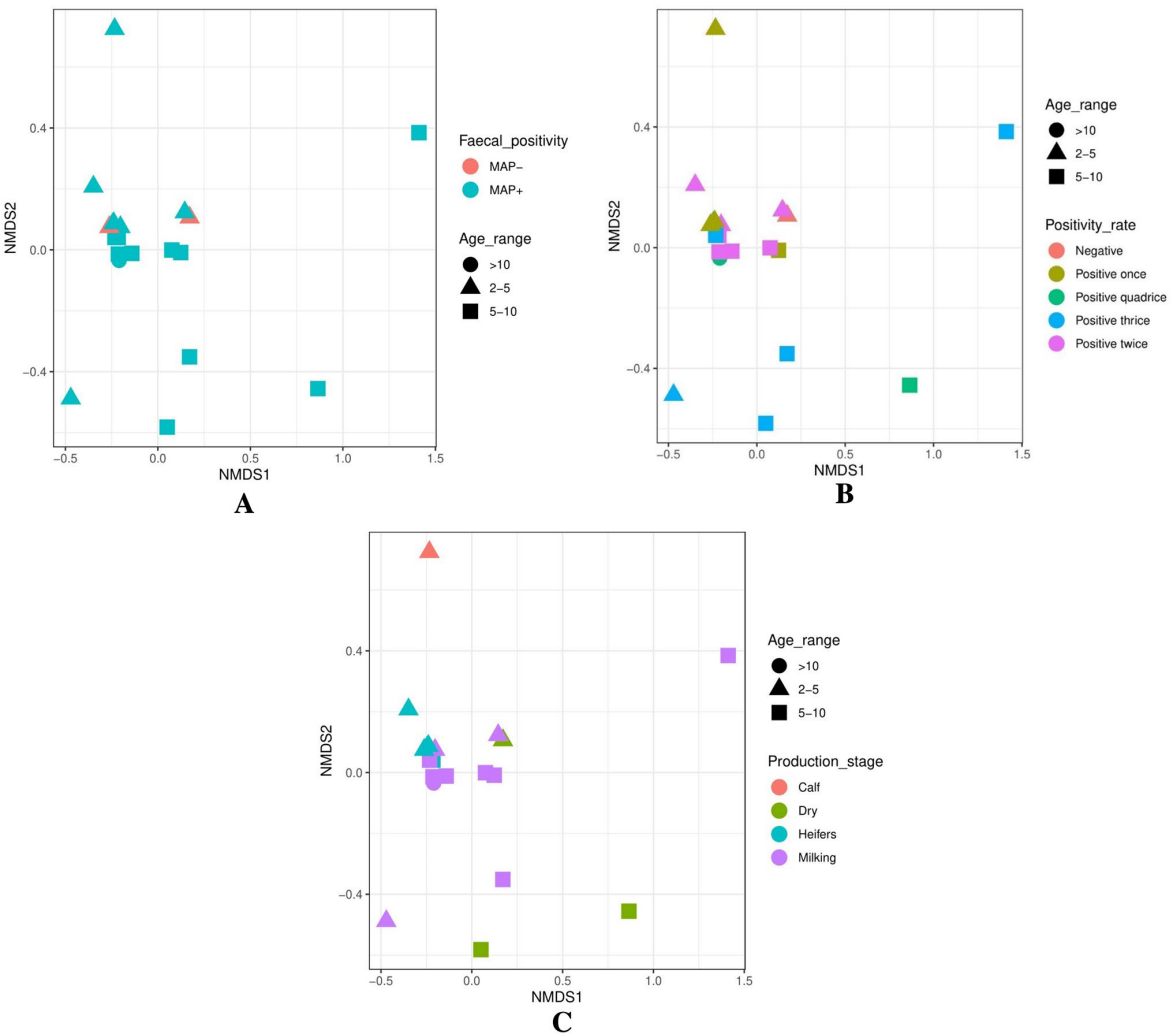
**a** Diversity of bacterial communities in the faeces of animals investigated for *Mycobacterium avium* subsp. *paratuberculosis* (MAP) measured by non-metric multidimensional scaling (NMDS) for animals at various age range and different MAP DNA positivity in faeces. **b** Diversity of bacterial communities in the faeces of animals investigated for *Mycobacterium avium* subsp. *paratuberculosis* (MAP) measured by non- metric multidimensional scaling (NMDS) for animals at various age and different frequency of MAP DNA detection in faeces. **c** Diversity of bacterial communities in the faeces of animals investigated for *Mycobacterium avium* subsp. *paratuberculosis* (MAP) measured by non- metric multidimensional scaling (NMDS) for animals at various age and different production status

The distance between samples, the beta diversity, ordinated by NMDS by age group and other categorization criteria; (Fig. 1) revealed that the community structure varied with age group rather than the other factors (Fig. 5a–c).

The ARA of the different taxa varied between the animals at different statuses of production, (Supplementary File 2). In all groups, the major phyla were firmicutes and bacteroidetes, while proteobacteria showed an ARA of < 1%. However, the noticeable findings were that the highest ARA of firmicute (95.8%) was in the animals at the dry stage and they had the lowest ARA of bacteroidetes (3.6%). In contrast, heifers had the lowest ARA of firmicutes (69.4%) and highest ARA of bacteroidetes (27.8%). The third abundant phylum differed: tenericutes in heifers (ARA = 1%) and proteobacteria in the dry cows (ARA = 0.36%).

Families with ARA > 1% were 12 in heifers and 8 in the dry cow groups. In heifers, *Oscillospiraceae* and *Rikenellaceae* represented ~ 50% of the families, while in the dry cows > 60% of total families were represented by *Planococcaceae* and *Streptococcaceae*.

At the genus level, the composition of heifers was more diverse, where 20 genera showed ARA > 1% with 6 genera constituting ~ 50% and the most abundant genera were *Alistipes* and *Acetivibrio*. However, in the dry cow group 11 genera showed ARA > 1%, and only two genera (*Kurthia* and *Streptococcus*) were dominating by 60.7% ARA.

**Fig. 6:**
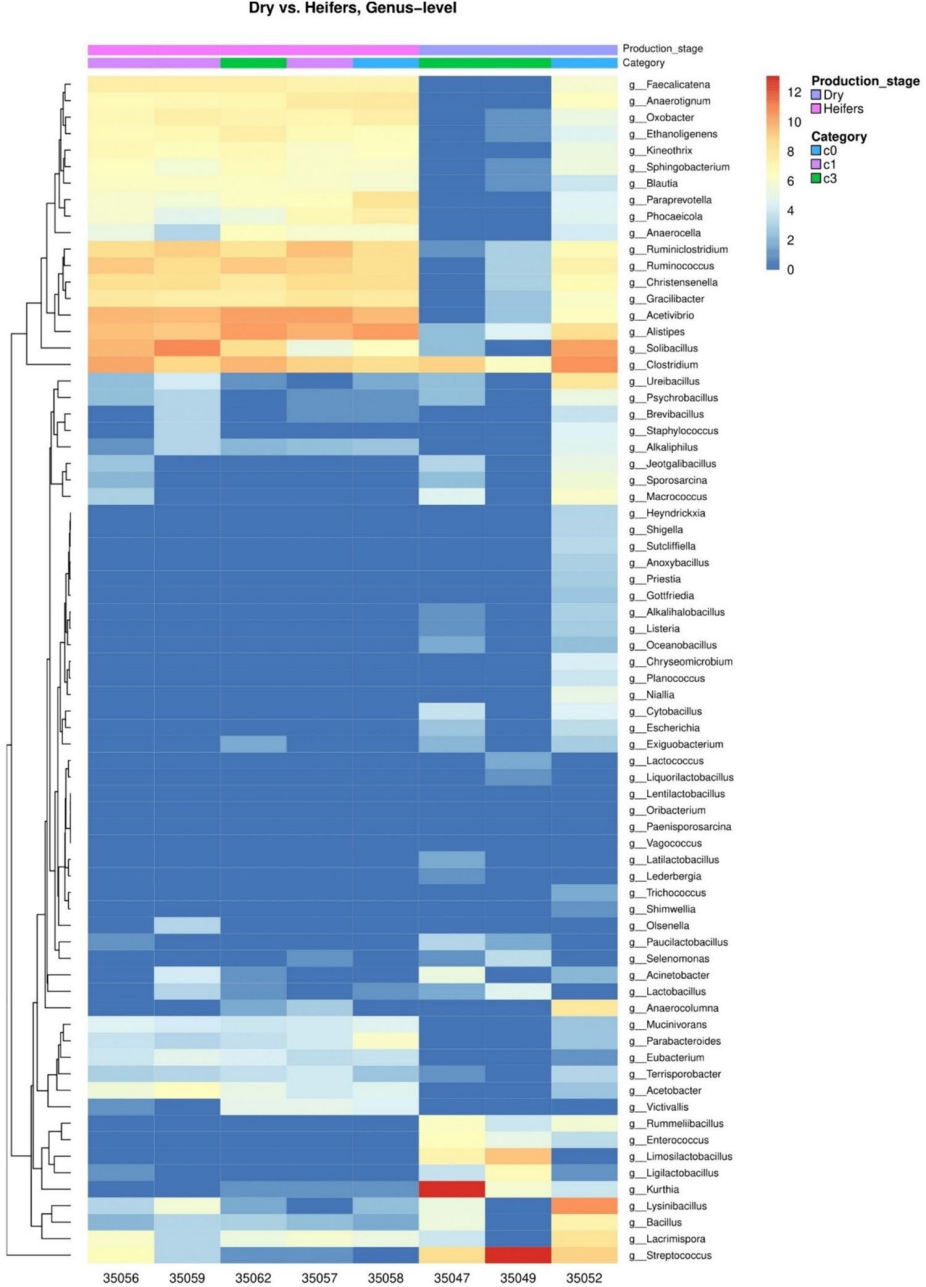
Heatmap showing the differential abundance of the significantly (*p* < 0.05) different genera in the faeces of heifers and dry cows in a dairy farm with paratuberculosis

A total of 72 genera were significantly different in abundance between dry cows and heifers (Fig. 6). Besides, some genera followed a pattern of ARA with production status; *Solibacillus*, *Bacteroides* and *Lactobacillus* showed decreasing ARA in the order: calf, heifer, lactating and dry cows. On the other hand, ARA of *Clostridium* and *Streptococcus* was increasing with production status.

Similarly, the difference at the species level between heifers and the dry was obvious: 15 species accounted for 50% of all those assigned in heifers, while in dry cows only two species, *Kurthia massiliensis* and *Sterptococcus lutetiensis*, represented ~ 55% of all species in the group. Also, in the heifers, species that showed > 1% ARA were 27, while in the dry cows they were only 8 species.

## Discussion

Infection with MAP induces gut inflammation and possibly changes the microbiome (Matthews et al. 2021). This study was designed to investigate the potential effect of MAP on the gut microbiome of cattle. To achieve this goal, a farm with established paratuberculosis was selected for the study. The animals were tested five times to identify the stage of the infection and the MAP shedding status in each animal. In general, the results showed variations in the shedding status with only two animals that were consistently negative for MAP faecal shedding. The faecal microbiome of the animals was analysed according to MAP positivity, faecal shedding, and animal factors, the latter two showed clues to having impact on the microbiome.

The presence of one infected case in a farm poses source of infection for the herd mates (Fecteau 2018; Garcia and Shalloo 2015; Rathnaiah et al. 2017). In the present case, the clinical diagnosis was confirmed by isolation of MAP. According to the iceberg phenomena, with every advanced case of PTB there will be many cases at different stages of the disease (Matthews et al. 2021; Fecteau and Whitlock 2010; Davis and Park 2018; Okuni et al. 2020). When the apparently healthy herd mates were sampled with the last clinical case, they were seronegative, however, MAP was isolated from one of them. This necessitated the use of various tests and multiple sampling, as the specificity and sensitivity of each test varies with the stage of the disease (Garcia and Shalloo 2015).

The sampling spanned 10 months, started monthly for 4 months then after 6 months a fifth samples set was collected. The monthly sampling for 4 months was chosen based on an observation reported in a previous study (Mortier et al. 2014), where MAP shedding was detected twice within 4 months period following the first culture positive from accidently infected calf (natural infection).

The detection rate for anti-MAP antibodies in serum and in milk was 22.7 and 21.4%, respectively. This close performance of ELISA in milk and serum has been reported in previous studies (Sweeney et al. 2012; Faruk et al. 2020). But the sampling is affected by production status (dry period), as in our study complete sample sets were obtained from 7 out of 14 lactating cows. However, those who were ELISA positive in milk became positive for DNA in faeces in the next sampling, i.e. antibodies were indicative for faecal shedding as has already been documented (Imada et al. 2020).

In the faeces MAP DNA was detected in 90.9% of the animals in all age groups. These animals were frequently exposed to an increasing and/or high infection pressure which could overcome the age-related resistance (Fecteau 2018). As a result, MAP could be detected in the faeces, either as passive (Kralik et al. 2014) or active shedding (Fecteau 2018). MAP faecal shedding in naturally infected calves < 3 months age has already been reported (Wolf et al. 2015), the same as the case of the single calf in this study. Despite the high infection pressure in the farm, two cows were consistently negative for MAP DNA in the faeces by both assays. Either they were at the subclinical stage of the disease, which could last for 2–10 years (Fecteau 2020), or they might be resistant to the infection and would likely contain it; in both cases, faecal shedding might be low and undetectable, or intermittent.

None of the animal factors (age, milk production, body condition, and history of PTB) had significant correlation with MAP positivity, however, the results can be taken with some caution because of the small sample size which might cause statistical random error.

Transitions in immune reactions to MAP infection and the consequent production of antibodies and commencement of shedding is not fully understood (Mallikarjunappa et al. 2021). The interactions between the immune system, genetics and microbiome are likely to play roles in infectious diseases. The development of the immune system is largely affected by the microbiome (Zhang et al. 2021). Animal’s genetics control the susceptibility to infections and guide microbiome composition (Li et al. 2019). On the other hand, gut microbiota regulates local inflammation (Akhtar et al. 2022) therefore, it could account for differences in the fate of the infection.

In the current investigation, firmicutes and bacteroidetes were dominating the faecal microbiome of the animals, the same as has been reported before (Clemmons et al. 2019; Dias and Ametaj 2017; Aricha et al. 2021; Alipour et al. 2018; Wang et al. 2022; Dill-McFarland et al. 2017). However, the abundance reached 99.23% with median 77.3% in firmicutes, and 80% with median 19.6% in bacteroidetes. Apart from MAP infection, temperature was found to affect faecal microbial composition; firmicutes abundance could increase in the tropical conditions (Zhang et al. 2022), such as those in the Sudan. In consistence with this, half of the animals investigated in the present study showed high abundance of firmicutes, especially in the group of dry cows, where it reached 95.8%. The abundance of proteobacteria was in range 0.01–5.96 with median 0.29. This was in accordance with the small contribution of proteobacteria on the faecal microbiome of adult cows reported earlier (Dias and Ametaj 2017). Another interpretation could be the effect of temperature as the noted gradual decrease in abundance with the increasing temperature (Zhang et al. 2022). The fact that microbiome composition is multifactorial could account for these inconsistences.

There was an obvious difference between samples in the current observations related to age which was also apparent before in the bacterial community of different age groups (Dill-McFarland et al. 2017).

Except for *Ruminococcaceae*, all families that were commonly found as the most abundant taxa in the faeces of adult cows (Dias and Ametaj 2017; Aricha et al. 2021; Alipour et al. 2018; Zhao et al. 2022; Dill-McFarland et al. 2017; Deng et al. 2019), such as *Bacteroidaceae* and *Lachnospiraceae*, were also assigned in the animals in the current investigation. Besides, these families were among the top 14 and 16 abundant families in individual animal and animal groups, respectively. Previous investigations on the possible dysbiosis predisposed by MAP showed inconsistent findings (Kaevska et al. 2016; Fecteau et al. 2016; Derakhshani et al. 2016), though, the decreased abundance of *Ruminococcaceae* was the only shared feature, the same as in this investigation. Members of the family *Ruminococcaceae* were distinguished as degrader of plant materials (Biddle et al. 2013).

The implication of MAP in dysbiosis was based on the infection- attributed local inflammation (Matthews et al. 2021). Sex is another factor that was found to affect an animal’s response to MAP infection (Karunasena et al. 2014). Also, it can affect the microbiome composition (Hussain et al. 2021; Nuriel-Ohayon et al. 2019; Wu et al. 2022). Age and puberty are among the factors that would affect the microbiota; however, the study by Guo et al. (2020) compared the microbiota in gut segments (rumen, abomasum, duodenum and rectum) and revealed that the diversity of bacterial contents of the samples obtained from the rectum, which are almost the same as the samples in our study, reached a plateau phase (no obvious difference was observed) before 50 weeks’ age, (Guo et al. 2020), i.e., before puberty.

The Bray–Curtis dissimilarity measure revealed a significant difference between heifers and dry cows. Previously, a difference in host response to MAP infection between male and female was observed and has been linked to gut microbiota (Karunasena et al. 2014; Karunasena et al. 2014). One of the major physiological differences between heifers and dry cows is the type of dominant reproductive hormone(s). These hormones are known potential factor that would change the microbial content in the gut of healthy animals (Hussain et al. 2021; Nuriel-Ohayon et al. 2019). Pregnancy enhances enrichment of proteobacteria and actinobacteria (Hussain et al. 2021). An increased abundance of *Bifidobacterium* during pregnancy was noted and was attributed to progesterone (Nuriel-Ohayon et al. 2019). Probable similar effect of pregnancy on microbiota was noted in the present study, however, *Bifidobacterium* was not assigned in this study but enrichment of the phylum firmicutes and the genus *Streptococcus* were found in the group of the dry cows (pregnant) when compared to heifers (non-pregnant). Heifers are dominated by reproductive hormones other than progesterone. An investigation on the shifts in microbiota during oestrus synchronization revealed that many taxa were significantly correlated with the levels of the reproductive hormones throughout the cycle (Wu et al. 2022). They found that the family *Akkermansiaceae* and the related genus *Akkermansia* were significantly correlated with the hormones throughout the cycle (Wu et al. 2022), but this taxa was not among the 72 genera those were of significant difference in abundance between dry cows and heifers groups in this study. The dissimilarities noted in this study in taxa that would probably be affected by reproductive hormones point out additional influencers on the microbiome, more likely MAP.

The diet of the animals was not considered a factor that would influence the microbiome in this study. The study farm follows a traditional system of production, in which, with the exception of pre-weaned calves, animals are not strictly separated and they may receive more or less the same feed.

Despite the faecal microbial composition was notably differed with varying positivity rate, the dissimilarity analysis (Bray-Curtis measure) showed insignificant difference between of MAP+ and MAP−. However, this could be justified by the difference in number of animals representing each group (18 vs 2). It is noteworthy that in case of such endemic disease, these two (MAP−) animals cannot easily be found in farms where PTP is established. What is also important is to have the same control from the same farm, especially in microbiome studies.

Samples from four animals in the present study clustered together as having close faecal microbial composition (Fig. 2). These four animals were ELISA positive indicating the progression of the disease and they were faecal positive for MAP DNA in at least three visits. Most of the taxa diminished and they shared the decreased abundance of the families *Bacteroidaceae*, *Prevoltellaceae* and *Rikenellaceae*, compared with the other animals, as has been noted before in a MAP-positive group (Fecteau et al. 2016). Further, two of the four animals showed the lowest Shannon (richness) and Pielou (evenness) indices. The MAP DNA positivity rate may not account alone for this difference. Neither all seropositive animals nor those with same frequency of MAP DNA positivity clustered with these four animals. The positivity rate by ELISA or DNA detection may or may not reflect the stage of the disease. When the macrophages fail to control the multiplication of phagocytosed MAP cells; they rupture which is associated within the production of antibodies (Nielsen 2014). Besides, shedding of MAP in the faeces could be passive (Kralik et al. 2014), i.e., not related to the infection. However, when it occurs in an increasing manner and accompanied by detectable antibodies, it would be indicative for disease progression. Though, the influence of MAP on the microbiome possibly differs in animals at different stages of disease progression.

The fermentation of indigestible fibres by gut microflora and production of SCFAs is an example of microbiome-host mutualism (Clemente et al. 2018; Al Bander et al. 2020; Akhtar et al. 2022). Also, gut microbes regulate host’s physiology while they are affected by its physiological state (Litvak et al. 2018). They protect against inflammation, which in turn would cause dysbiosis (Kiely et al. 2018). In fact, the microbiome is highly dynamic and complex in nature; its composition and function are multifactorial. *Streptococcus* spp., as an example, produce SCFAs as by-product of digestion of complex carbohydrates (Akhtar et al. 2022) and at the same time, produce toxic compounds by fermenting proteins (Brüssow 2013). Therefore, the co-existence and relative abundance of taxa along with diet and metabolic machinery of the host determine to a large extent the significance of a specific taxon within a niche. Hence, the results of this study cannot be interpreted without considering all these facts about host-microbiome interactions.

The current observations suggest that the faecal microbiome in animals with MAP infection is affected by both the pathogen and the production status/physiological state rather than by MAP alone. Further investigations would provide more insights into the individual and/ or collective effects of factors other than production status such as age, diet, and weather conditions with MAP infection on the faecal microbiome as well as the consequent effect on the fate of the infection. As well, the extent of the progression of the disease would likely affect the microbial content of the faeces more than mere presence of MAP.

### Supplementary Information

Below is the link to the electronic supplementary material.

Supplementary file1 (DOCX 35 KB)

Supplementary file2 (XLSX 45 KB)

## Data Availability

The sequencing datasets generated during and analysed for the current study are available online at https://zenodo.org/records/10458438.
