# Supplementary material for: Faecal microbial diversity in a cattle herd infected by Mycobacterium avium subsp. paratuberculosis: a possible effect of production status
Source: World J Microbiol Biotechnol. 2024 Jul 22;40(9):276. doi: 10.1007/s11274-024-04080-1 (PMC11263420; doi:10.1007/s11274-024-04080-1)
Supplement: Supplementary file 1 — Supplementary file1 (DOCX 35 KB) [file 11274_2024_4080_MOESM1_ESM.docx]

Faecal microbial diversity in cattle herd infected by *Mycobacterium* *avium* subsp. *paratuberculosis*: a possible effect of production status

**World Journal of Microbiology and Biotechnology**

Wisal A. Elmagzoub^1,2^, Sanaa M. Idris^1,3,^ ^ǂ^, Marwa H. E. Elnaiem^4,^ ^ǂ^, Mohamed E. Mukhtar^5^, ElSagad Eltayeb^6^, Sahar M. Bakhiet^7^, Julius B Okuni^8^, Lonzy Ojok^9,^ , Sulieman M. El Sanousi ^10,^ Ahmed Abd El Wahed^11*^, Ahmed A. Gameel^3^, Kamal H. Eltom^1*^

1 Unit of Animal Health and Safety of Animal Products, Institute for Studies and Promotion of Animal Exports, University of Khartoum, Shambat 13314, Khartoum North, Sudan; wisalelmagzoub@gmail.com (W.A.E.), sanaaidris15@gmail.com (S.M.I.), keltom@daad-alumni.de (K.H.E.)

2 Department of Biology and Biotechnology, College of Applied and Industrial Sciences, University of Bahri, Khartoum North, Sudan; wisalelmagzoub@gmail.com (W.A.E.)

3 Department of Pathology, Faculty of Veterinary Medicine, University of Khartoum, Shambat 13314, Khartoum North, Sudan; sanaaidris15@gmail.com (S.M.I.), aargameel@hotmail.com (A.A.G.)

4 Department of Botany and Agricultural Biotechnology, Faculty of Agriculture, University of Khartoum, Shambat, 13314, Khartoum North, Sudan, marwahat@gmail.com (M.H.E.E)

5 Department of Agricultural Extension and Rural Development, Faculty of Agriculture, University of Khartoum, Shambat 13314, Khartoum North, Sudan; elgadidwas@yahoo.com (M.E.M).

6 Faculty of Medicine, Al Neelain University/ Ibn Sina Specialised Hospital, Alamarat 12217, Street 17 -21 Khartoum, Sudan; sagadgady@yahoo.com (E.E.)

7 Department of Molecular biology, Institute of Endemic diseases, Sudan, Saharbakhiet@iend.org (S.M.B.)

8 College of Veterinary Medicine, Animal Resources and Biosecurity (COVAB), Makerere University, P. O. Box 7062, Kampala, Uganda; jbok@covab.mak.ac.ug (J.B.O.), lonzyo@yahoo.com (L.O.)

9 Department of Pathology, Faculty of Medicine, Gulu University, P.O.Box 166, Gulu, Uganda; lonzyo@yahoo.com (L.O)

10 Department of Microbiology, Faculty of Veterinary Medicine, University of Khartoum, Shambat 13314, Khartoum North, Sudan, sanousi07@gmail.com (S.M.E).

11 Institute of Animal Hygiene and Veterinary Public Health, Faculty of Veterinary Medicine, University of Leipzig, An den, Tierkliniken 1, D-04103 Leipzig, Germany; ahmed.abd_el_wahed@uni-leipzig.de (A.A.E.W.)

ǂ equally contributed

*Correspondence: kamal@daad-alumni.de; ahmed.abd_el_wahed@uni-Leipzig.de; Tel.: +249-91-028-9916 (K.H.E.); +49-3419-738-153 (A.A.E.W.)

**Tables**

Table 1: Detection of anti-*Mycobacterium avium* subsp. *paratuberculosis* antibodies in milk and serum of animals in the study farm

| Animal Code | Production status | Serum ELISA | | | | | Milk ELISA | | | | |
| --- | --- | --- | --- | --- | --- | --- | --- | --- | --- | --- | --- |
|  |  | V1 | V2 | V3 | V4 | V5 | V1 | V2 | V3 | V4 | V5 |
| 35041 | Milking | - | - | - | - | - | - | - | - | - | - |
| 35042 | Milking | - | - | - | - | - | - | - | D | + | - |
| 35043 | Milking | - | - | - | - | - | - | - | - | - | - |
| 35044 | Milking | - | - | - | - | - | - | - | - | + | - |
| 35045 | Milking | - | - | - | - | - | - | - | - | - | - |
| 35047 | Milking | + | - | - | - | - | - | - | - | - | D |
| 35048 | Milking | + | NP | + | + | + | + | D | D | + | + |
| 35049 | Dry | + | - | - | + | - | - | - | - | - | D |
| 35050 | Milking | - | - | - | - | - | - | - | - | - | - |
| 35051 | Milking | + | - | - | - | - | + | D | D | - | - |
| 35052 | Dry | - | - | - | - | - | - | - | - | - | D |
| 35053 | Milking | - | - | - | - | - | - | - | - | - | - |
| 35054 | Milking | - | - | - | - | - | - | - | - | D | - |
| 35055 | Milking | - | - | - | - | - | H | H | H | H | H |
| 35056 | Heifer | + | - | - | - | - | H | H | H | H | H |
| 35057 | Heifer | - | - | - | - | - | H | H | H | H | H |
| 35058 | Heifer | - | - | - | - | - | H | H | H | H | H |
| 35059 | Heifer | - | - | - | - | - | H | H | H | H | H |
| 35060 | Heifer | - | - | - | - | Died | H | H | H | H | Died |
| 35061 | Heifer | - | - | - | - | - | H | H | H | H | H |
| 35062 | Calf | - | - | - | - | - | H | H | H | H | H |
| 35065 | Calf | NA | - | - | - | - | NA | C | C | C | C |
| 350100 | Milking | - | - | - | - | - | + | D | D | D | - |

Key: C: Calf, D: Dry, H: Heifer, NA: Not Applicable NP: Not Provided

Table 2: Detection of *Mycobacterium avium* subsp. *paratuberculosis* DNA in faeces and milk of animals in the study farm

| Animal code | Production status | Faecal DNA | | | | | | Milk DNA | | | | |
| --- | --- | --- | --- | --- | --- | --- | --- | --- | --- | --- | --- | --- |
|  |  | V1 | V2 | V3 | V4 | V5 | V1 | | V2 | V3 | V4 | V5 |
| 35041 | Milking | + | + | - | - | - | + | | - | + | + | - |
| 35042 | Milking | + | + | - | - | - | - | | + | D | + | - |
| 35043 | Milking | + | - | + | - | - | + | | - | - | - | - |
| 35044 | Milking | + | + | + | - | - | + | | - | + | - | - |
| 35045 | Milking | + | + | + | - | - | - | | - | - | - | - |
| 35047 | Dry | + | - | - | + | - | - | | - | + | - | D |
| 35048 | Milking | + | + | + | - | - | + | | D | D | - | + |
| 35049 | Dry | - | + | + | - | - | + | | + | + | - | - |
| 35050 | Milking | + | + | - | + | - | - | | - | + | - | D |
| 35051 | Milking | - | + | - | - | - | + | | D | D | + | - |
| 35052 | Dry | - | - | - | - | - | - | | - | + | + | - |
| 35053 | Milking | - | + | - | - | - | - | | - | + | + | - |
| 35054 | Milking | - | - | + | - | - | + | | - | - | D | - |
| 35055 | Milking | - | + | + | - | - | H | | H | H | H | H |
| 35056 | Heifer | - | - | - | + | - | H | | H | H | H | H |
| 35057 | Heifer | - | - | - | - | - | H | | H | H | H | H |
| 35058 | Heifer | + | - | - | - | - | H | | H | H | H | H |
| 35059 | Heifer | - | - | - | + | - | H | | H | H | H | H |
| 35060 | Heifer | - | + | - | - | Died | H | | H | H | H | Died |
| 35061 | Heifer | - | + | - | - | - | H | | H | H | H | H |
| 35062 | Calf | + | + | + | - | - | H | | H | H | H | H |
| 35065 | Calf | NA | + | - | - | - | NA | | C | C | C | C |
| 350100 | Milking | - | + | - | + | - | - | | D | D | D | + |

Key: C: Calf, D: Dry, H: Heifer, NA: Not Applicable
